# Medical Imaging-Based Kinematic Modeling for Biomimetic Finger Joints and Hand Exoskeleton Validation

**DOI:** 10.3390/biomimetics10100652

**Published:** 2025-10-01

**Authors:** Xiaochan Wang, Cheolhee Cho, Peng Zhang, Shuyuan Ge, Jiadi Chen

**Affiliations:** 1 Department of Design, Kyungpook National University, Daegu 41566, Republic of Korea; daegu9238@gmail.com (X.W.); chery@knu.ac.kr (C.C.); 2 School of Arts and Design, Yanshan University, Haigang District, Qinhuangdao 066000, China; zp18713581063@gmail.com; 3 Department of Electrical and Electronic Engineering, Kyungpook National University, Daegu 41566, Republic of Korea; gsy8933@gmail.com

**Keywords:** hand rehabilitation exoskeleton, instantaneous center of rotation (ICOR), biomimetic kinematic pair, human–machine compatibility

## Abstract

Hand rehabilitation exoskeletons play a critical role in restoring motor function in patients with stroke or hand injuries. However, most existing designs rely on fixed-axis assumptions, neglecting the rolling–sliding coupling of finger joints that causes instantaneous center of rotation (ICOR) drift, leading to kinematic misalignment and localized pressure concentrations. This study proposes the Instant Radius Method (IRM) based on medical imaging to continuously model ICOR trajectories of the MCP, PIP, and DIP joints, followed by the construction of an equivalent ICOR through curve fitting. Crossing-type biomimetic kinematic pairs were designed according to the equivalent ICOR and integrated into a three-loop ten-linkage exoskeleton capable of dual DOFs per finger (flexion–extension and abduction–adduction, 10 DOFs in total). Kinematic validation was performed using IMU sensors (Delsys) to capture joint angles, and interface pressure distribution at MCP and PIP was measured using thin-film pressure sensors. Experimental results demonstrated that with biomimetic kinematic pairs, the exoskeleton’s fingertip trajectories matched physiological trajectories more closely, with significantly reduced RMSE. Pressure measurements showed a reduction of approximately 15–25% in mean pressure and 20–30% in peak pressure at MCP and PIP, with more uniform distributions. The integrated framework of IRM-based modeling–equivalent ICOR–biomimetic kinematic pairs–multi-DOF exoskeleton design effectively enhanced kinematic alignment and human–machine compatibility. This work highlights the importance and feasibility of ICOR alignment in rehabilitation robotics and provides a promising pathway toward personalized rehabilitation and clinical translation.

## 1. Introduction

Hand rehabilitation plays a vital role in the functional recovery of patients with stroke, peripheral nerve injury, and hand trauma. Hand exoskeletons have attracted significant attention due to their ability to provide controlled assistance and repetitive training [1,2,3]. Most existing devices rely on rigid linkages or soft tendon-driven structures and primarily focus on flexion–extension assistance [4,5,6]. However, the real kinematics of finger joints are far more complex than simple hinge-like motion. Instead, finger joints exhibit a rolling–sliding mechanism, leading to an Instantaneous Center of Rotation (ICOR) that continuously drifts during flexion [7,8,9,10]. Ignoring this phenomenon results in misalignment between the exoskeleton and the biological joint, producing additional shear and compressive stresses, which compromise user comfort and safety [11]. Prior work has quantitatively linked human–exoskeleton joint misalignment to higher interface pressures and lower user comfort. Andrade et al. reported that a frontal-guard ankle–foot orthoses exhibited smaller vertical misalignment peaks (1.37 ± 0.90 cm vs. 2.95 ± 0.64 cm) and lower median interface pressures (0.39–3.12 kPa vs. 3.19–19.78 kPa) than a commercial H2-AFO, together with higher perceived comfort (*p* < 0.05) [12].

In terms of joint modeling, early studies estimated the joint center of rotation using optical motion capture or anatomical assumptions [7,13,14]. More recently, medical imaging techniques such as CT and MRI have enabled high-precision reconstruction of bone geometry and joint kinematics. Cerveri demonstrated the feasibility of estimating the ICOR from reconstructed 3D models [15]. Nevertheless, most existing methods focus on discrete postures and lack continuous kinematic modeling, which limits their applicability to exoskeleton design. Thus, developing an imaging-based continuous modeling approach is critical for biomimetic and personalized exoskeleton development.

Rigid linkage-based hand exoskeletons are widely studied due to their mechanical stability and efficient power transmission [16,17,18]. Sarac et al. introduced an underactuated linkage-based mechanism that automatically adapts to finger length variations [19]. Carbone et al. designed a two-DOF driving mechanism to synchronize multiple finger joints [20]. Talat et al. applied a series elastic actuator to improve compliance while maintaining control accuracy [21]. Dragusanu et al. proposed a differential gear mechanism for underactuated designs, simplifying the drive system while ensuring lightweight performance [22]. Although these studies advanced linkage-based designs, most remain restricted to flexion–extension motions and fail to address lateral finger movements.

Regarding multi-DOF exoskeletons, Fu et al. developed a system capable of independently actuating MCP and PIP joints but limited to sagittal plane motions [23]. Hyunki et al. presented the Exo-Glove, which supports flexion and partial abduction using soft tendons, but precision was limited by the compliant structure [24]. Overall, multi-DOF hand exoskeletons are still either confined to sagittal plane flexion/extension or rely on soft actuators, with little exploration of rigid linkage designs that simultaneously support flexion and lateral abduction/adduction.

Therefore, current research gaps can be summarized as follows: (1) insufficient continuous modeling of finger ICOR, leading to persistent misalignment issues; (2) limited multi-DOF support in rigid linkage exoskeletons, particularly lacking abduction/adduction capability; and (3) the absence of an integrated framework combining medical imaging-based modeling, biomimetic mechanism design, and experimental validation.

To address these challenges, this paper proposes an imaging-driven “Instant Radius Method” to continuously model finger ICOR trajectories and establishes an equivalent ICOR model for engineering design. Based on this, a novel three-loop, ten-linkage exoskeleton is designed, which enables both flexion–extension and lateral abduction/adduction motions, achieving a total of ten DOFs across five fingers. A biomimetic revolute pair is introduced to align the exoskeleton’s motion center with the biological ICOR. Finally, kinematic trajectory analysis and wearable pressure tests are conducted to validate the human–machine compatibility and comfort of the proposed design.

The main contributions of this work are as follows: (1) proposing the imaging-driven Instant Radius Method for continuous ICOR modeling; (2) establishing an integrated design framework combining equivalent ICOR with rigid linkage structures; and (3) developing and validating a lightweight, multi-DOF biomimetic exoskeleton capable of supporting both flexion and abduction/adduction, thereby enhancing rehabilitation effectiveness and user comfort. This paper emphasizes imaging and mechanism design, while mechatronics/control will be detailed in a follow-up article.

## 2. Materials and Methods

### 2.1. Establish a Kinematic Model of Finger Joint

#### 2.1.1. Acquisition of Finger Joint Kinematic Data Based on Medical Imaging

**Figure 1:**
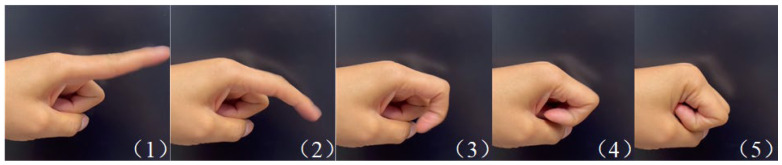
Key frames of finger flexion process.

To accurately capture the kinematic characteristics of finger joints during flexion and extension, the index finger was selected as the primary subject, with particular focus on the metacarpophalangeal (MCP), proximal interphalangeal (PIP), and distal interphalangeal (DIP) joints. First, a high-speed camera was used to record the natural flexion–extension motion of the index finger, which lasted approximately 400 ms. The motion sequence was evenly divided into five key frames, each separated by approximately 80 ms, as shown in Figure 1. These five key postures were subsequently used as references for CT scanning, with each posture requiring one CT scan. Consequently, each volunteer underwent five local CT scans of the index finger.

To ensure representativeness, a total of ten healthy adult volunteers were recruited. The cumulative radiation dose from five scans per subject remained within clinically accepted safety limits [25,26,27,28]. As each scan lasted around 2 min, the finger had to remain immobile throughout the process. Considering that metallic or high-density fixation materials may generate artifacts in CT images and interfere with bone segmentation, a polymer plaster splint was used as the fixation device. The splint, primarily composed of fiberglass, is soft and towel-like before hardening and becomes rigid within 5 min after water activation. Thus, the splint was rapidly shaped into the desired posture before hardening and subsequently secured to the subject’s finger with bandages. This method ensured effective immobilization without introducing imaging artifacts.

The CT scanning parameters were set to 120 kV tube voltage, 525 mA tube current, and a slice thickness of 0.625 mm. Each subject was scanned sequentially according to the predefined posture sequence, resulting in five sets of CT data representing different key motion states. All data were exported in DICOM format for subsequent kinematic modeling. The experimental protocol has been approved by the Ethics Committee of Chengde Medical University (Approval Number: 2025002), and all participants have signed the informed consent form.

**Figure 2:**
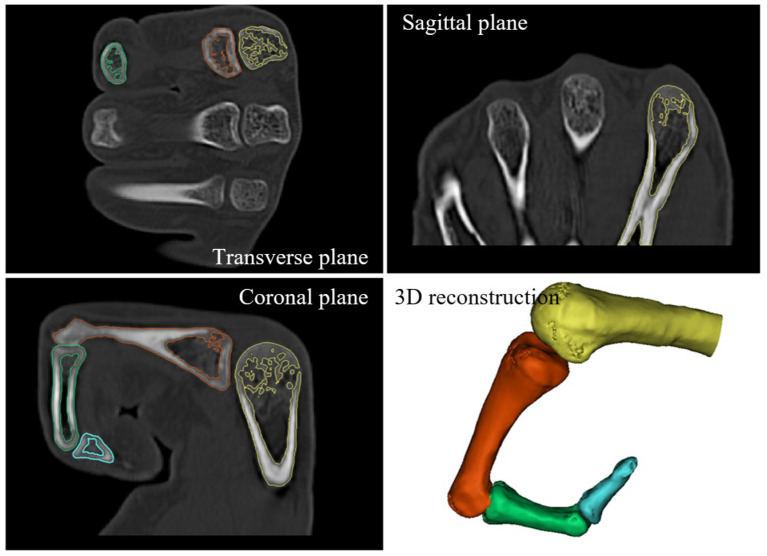
CT imaging processing results of index finger (The metacarpal bone is yellow, the proximal phalanx is orange, the middle phalanx is green, and the distal phalanx is blue).

For data preprocessing, the DICOM files were imported into Mimics software (version 14.0) for three-dimensional reconstruction. Sagittal, coronal, and transverse grayscale images were first generated from the original CT slices. Bone structures were then segmented from soft tissues and skin using thresholding and region-growing methods, and masks were established [29]. Subsequently, the skeletal structure was further subdivided into the metacarpal, proximal phalanx, middle phalanx, and distal phalanx, resulting in independent 3D bone models, as shown in Figure 2. These processed models not only provided clear anatomical boundaries for the Instant Radius Method (IRM)-based modeling but also laid the anatomical foundation for the design of biomimetic kinematic pairs in subsequent stages.

#### 2.1.2. Data Processing and Instant Radius Method Modeling

After acquiring CT data of finger bones under multiple postures and completing preprocessing, further modeling of joint motion characteristics was required. Analysis of the contact behavior between articular surfaces revealed that finger joints do not rotate around a fixed center. Instead, they exhibit hybrid rolling–sliding motion, where the instantaneous center of rotation (ICOR) continuously drifts during flexion. Determining the trajectory of this ICOR is therefore critical for constructing an accurate kinematic model of finger joints.

In biomechanics, existing approaches such as principal component analysis (PCA) and iterative closest point (ICP) algorithms have been used to locate the ICOR [30,31]. However, these methods are computationally intensive and generally provide only a few discrete ICOR points, which are insufficient for capturing continuous kinematic patterns. To overcome this limitation, this study proposes an ‘Instant Radius Method (IRM),’ which employs curve fitting of bone surface contact points combined with polar coordinate transformation to obtain continuous ICOR trajectories.

The principle of the IRM is as follows: when the phalangeal base undergoes hybrid rolling–sliding motion relative to the phalangeal head, the ICOR can be represented by a directed line segment pointing toward the contact point. When rolling and sliding directions are consistent, the ICOR lies on the line between the contact point and its curvature center; when inconsistent, the ICOR shifts accordingly. The sliding displacement is derived from the difference between the trajectories of the base and head contact curves, and the ratio of this displacement to the angular variation defines the length of the instant radius. The direction of the instant radius is determined by the normal vector at the contact point. Based on these definitions, the analytical expression of the instant radius can be formulated.

(1) $$R^{*}=i\left|\frac{d\delta }{d\theta }\right|$$

Here, $R^{*}$ is the instant radius, i is the unit normal vector of the contact curve at the current contact point, δ is the difference between the contact trajectories of the phalangeal base and the phalangeal head in this segment, and θ is the rotation angle of the phalangeal head in this segment.

**Figure 3:**
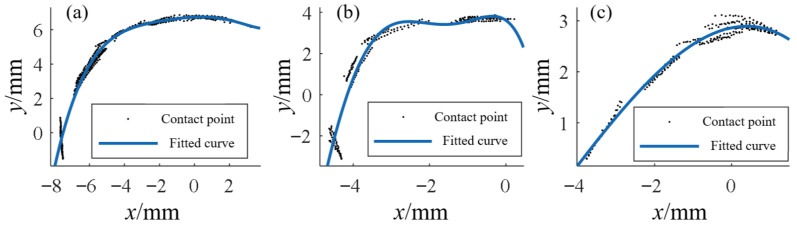
Fitted contact curves of articular surface contact points: (**a**) Metacarpal head; (**b**) Proximal phalanx head; (**c**) Middle phalanx head.

To obtain continuous ICOR trajectories, contact points extracted from CT images were first fitted into curves. Results showed that the head-side contact points exhibited strong regularity and were approximated by continuous functions, as shown in Figure 3, whereas the base-side contact points were more scattered and were approximated using piecewise functions. By combining the fitted curves with the arc-length integral formula, the modulus of the instant radius was derived.

Let the contact point equation of the phalangeal head be $y\;=h\left(x\right)$, and the contact point equation of the phalangeal base be $y\;=\;b\left(x\right)$. According to the arc-length integral formula, we obtain:

(2) $$d\delta =\sqrt{1+h^{\prime }{\left(x\right)}^{2}}dx-\sqrt{1+b^{\prime }{\left(x\right)}^{2}}dx$$

Since δ is not a function of θ, it is difficult to solve for $R^{*}$ by combining Equation (2) with Equation (1). Therefore, we attempt to transform $y\;=h\left(x\right)$ and $y\;=\;b\left(x\right)$ into polar coordinate equations, taking the phalanx axis as the polar axis to ensure the correct mapping of angle to contact point. Using the coordinate transformation formulas x=rcosθ and y=rsinθ, we can obtain $r_{h}\left(\theta \right)$ and $r_{b}\left(\theta \right)$. At this point:

(3) $$d\delta =\sqrt{r_{h}{\left(\theta \right)}^{2}+r_{h}^{\prime }{\left(\theta \right)}^{2}}d\theta -\sqrt{r_{b}{\left(\theta \right)}^{2}+r_{b}^{\prime }{\left(\theta \right)}^{2}}d\theta$$

By combining Equation (3) with Equation (1), we obtain:

(4) $$\left|R^{*}\right|=\sqrt{r_{h}{\left(\theta \right)}^{2}+r_{h}^{\prime }{\left(\theta \right)}^{2}}-\sqrt{r_{b}{\left(\theta \right)}^{2}+r_{b}^{\prime }{\left(\theta \right)}^{2}}$$

From Equation (4), the modulus function of the instant radius can be obtained. The direction i of the instant radius corresponds to the normal direction of points on $r_{h}\left(\theta \right)$ and $r_{b}\left(\theta \right)$ in the polar coordinate system, i.e., the negative reciprocal of the derivative at each point. Since $r_{h}\left(\theta \right)$ and $r_{b}\left(\theta \right)$ are in rigid contact, their tangents and normals at the contact point are identical. Therefore, only $r_{h}\left(\theta \right)$ is studied, and the directional function of i in polar coordinates can be expressed as:

(5) $$\alpha =\arctan(-\frac{1}{r_{h}^{\prime }(\theta )})$$

**Figure 4:**
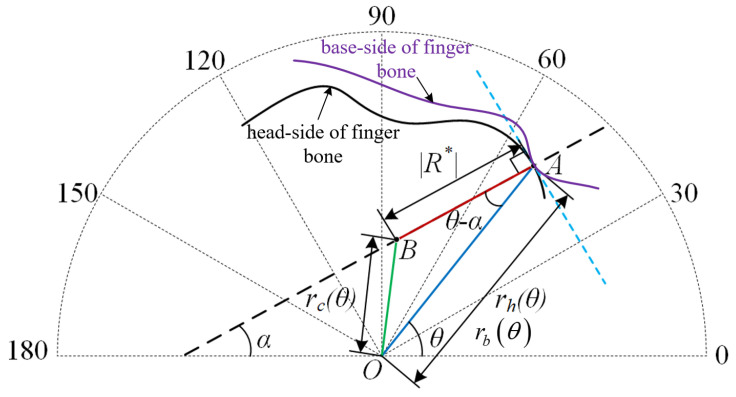
Determination of ICOR position in the polar coordinate system using the instant radius.

After obtaining the modulus and direction of the instant radius, the ICOR position in the polar coordinate system can be determined, as shown in Figure 4, where A is any contact point on the curve $r_{h}\left(\theta \right)$. The blue dashed line represents the tangent of $r_{h}\left(\theta \right)$ at point A, and the black dashed line represents the normal at point A.

Based on the geometric relationship in Figure 4, the cosine theorem can be used to derive the trajectory equation $r_{c}\left(\theta \right)$ of the ICOR in the polar coordinate system:

(6) $$r_{c}\left(\theta \right)={\left|R^{*}\right|}^{2}+r_{h}{\left(\theta \right)}^{2}-2\left|R^{*}\right|\cdot r_{h}(\theta )\cos\left(\theta -\alpha \right)$$

**Figure 5:**
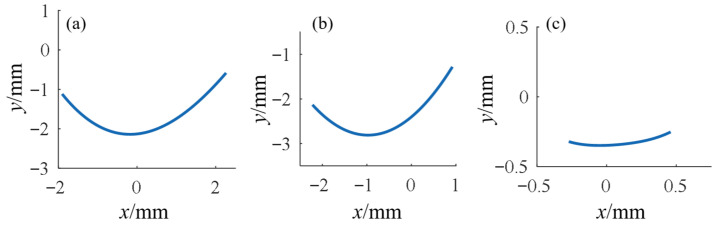
ICOR trajectories of finger joints: (**a**) MCP joint; (**b**) PIP joint; (**c**) DIP joint.

To subsequently obtain the kinematic model equations of finger joints in the Cartesian coordinate system, Equation (6) needs to be inversely transformed into $y_{c}\;=\;f\left(x_{c}\right)$, where $\left(x_{c},y_{c}\right)$ represents the ICOR coordinates. By taking the domain of the contact curve, the ICOR trajectory in the Cartesian coordinate system is obtained, as shown in Figure 5.

These trajectories not only revealed the intrinsic rolling–sliding characteristics of finger joint motion but also provided precise kinematic references for constructing equivalent ICOR models and designing biomimetic exoskeleton mechanisms.

### 2.2. Design of Biomimetic Kinematic Pairs for Finger Joints

After obtaining the ICOR trajectories of the MCP, PIP, and DIP joints, the next challenge was to translate these kinematic patterns into practical mechanical constraints for exoskeleton design. Because the real ICOR drifts during flexion–extension, conventional revolute joints with fixed centers of rotation inevitably cause misalignment between the exoskeleton and the biological finger, leading to additional soft tissue stress and friction. To address this issue, a biomimetic kinematic pair design based on the concept of an ‘equivalent ICOR’ was proposed in this study.

The equivalent ICOR is defined as an approximate rotation center derived from mathematical fitting and optimization of the continuous ICOR trajectory, such that the overall deviation between the simplified center and the real trajectory is minimized. In this work, the least-squares method was used to fit the ICOR trajectory, and the equivalent ICOR was determined by minimizing the sum of squared deviations. This approach ensures that the simplified model remains computationally tractable while preserving the essential kinematic characteristics of the finger joint.

**Figure 6:**
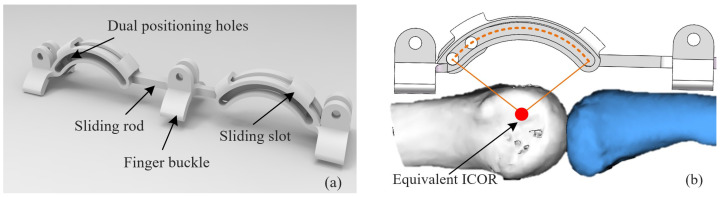
Design of a cross-type revolute joint: (**a**) Crossing-type revolute pair structure; (**b**) Following the equivalent ICOR.

To implement this concept mechanically, a ‘crossing-type revolute pair’ was designed. The geometric axis of this revolute pair coincides with the equivalent ICOR, and the linkage of the exoskeleton is connected to the external surface of the finger through a crossing connector, as shown in Figure 6. This configuration avoids the constraint limitations of planar revolute joints and ensures alignment of the exoskeleton’s rotation center with the biological ICOR. As a result, the exoskeleton achieves high kinematic conformity with the finger joints throughout the flexion–extension process, thereby reducing soft tissue load and improving both comfort and human–machine compatibility.

In terms of material selection and manufacturing, lightweight engineering plastics were adopted to balance strength and compliance. Flexible pads were incorporated at the joint interfaces to buffer local contact pressures. The resulting biomimetic kinematic pairs not only align with the kinematic features of human finger joints but also enhance structural safety and user comfort, forming the essential foundation for the overall exoskeleton design.

### 2.3. Lightweight Ten-DOF Hand Exoskeleton Design

After establishing the joint kinematic model and designing the biomimetic kinematic pairs, this study further proposes a lightweight ten-degree-of-freedom (DOF) hand rehabilitation exoskeleton. The exoskeleton follows a modular design, where each finger is actuated by an independent linkage mechanism to achieve flexion–extension. In addition, a lateral swinging mechanism is introduced at the MCP joint, enabling abduction and adduction in addition to flexion–extension. In this way, the five fingers collectively realize 10 DOFs, supporting common rehabilitation tasks such as grasping and releasing, as well as finger individuation and coordinated closing, thereby approximating natural hand movements.

For the flexion transmission system, a three-loop ten-linkage mechanism is proposed. The mechanism consists of nine moving links, denoted as $l_{1}$, $l_{2}$, $l_{3}$, …, $l_{9}$. Among them, $l_{1}$, $l_{2}$, $l_{5}$, $l_{7}$ contain intermediate joints, with front and rear segments denoted as $l_{x-1}$ and $l_{x-2}$. The frame length is represented by f. Three closed loops are formed: $L_{1}$, $L_{2}$, $L_{3}$. Specifically, loop $L_{1}$ is a five-bar mechanism (including one frame), loop $L_{2}$ is a six-bar mechanism, and loop $L_{3}$ is another five-bar mechanism.

**Figure 7:**
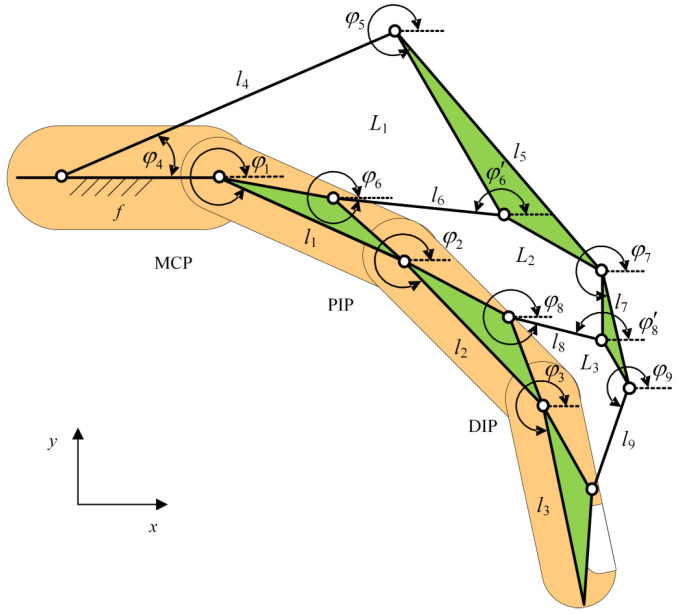
The exoskeleton mechanism in Lagrangian coordinates.

According to the principle of establishing a Lagrangian coordinate system, the positive x direction is defined as horizontal rightward, and the counterclockwise angle between each link and the x axis is defined as the positive angle. As shown in Figure 7, the mechanism contains 11 Lagrangian coordinates: $\varphi_{1}$, $\varphi_{2}$, …, $\varphi_{9}$, $\varphi_{6}^{\prime }$, $\varphi_{8}^{\prime }$. Since $\varphi_{6}^{\prime }$ is linearly dependent on $\varphi_{6}$, and $\varphi_{8}^{\prime }$ is linearly dependent on $\varphi_{8}$, there are effectively only nine independent coordinates, indicating that the mechanism is underactuated.

**Figure 8:**
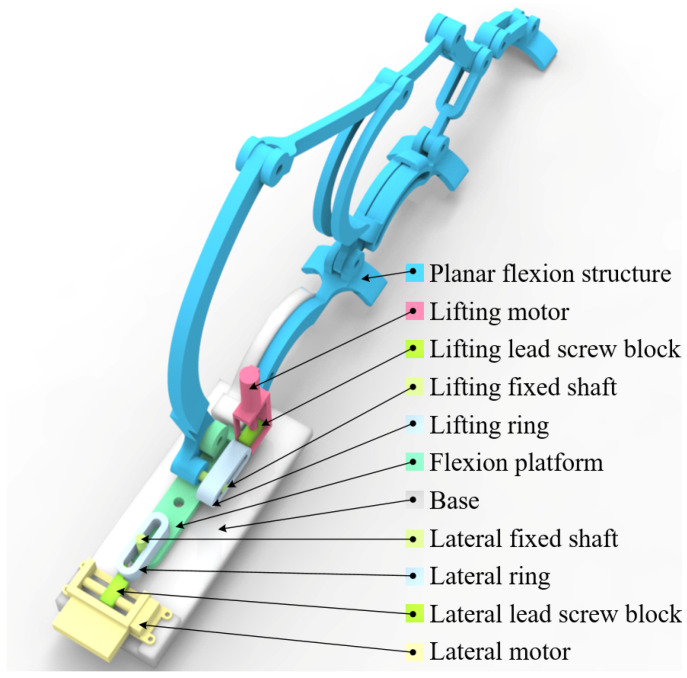
Exoskeleton structure of a single finger with two DOFs equipped with biomimetic kinematic pairs.

To ensure alignment with the ICOR of human joints, the revolute pairs at the MCP and PIP joints were replaced with the biomimetic kinematic pairs introduced in Section 2.2. Considering the small motion range and limited space at the DIP joint, a conventional revolute pair was retained there. Furthermore, to achieve abduction–adduction, a lateral swinging mechanism was added to the planar flexion system, expanding it into a spatial two-DOF mechanism. As shown in Figure 8, the MCP and PIP joints were equipped with biomimetic pairs, and the flexion platform was combined with a lateral swinging axle, enabling the entire flexion module to swing under lateral actuation. The flexion platform consists of a fixing hole, a flexion hinge hole, and a lateral fixation hole, serving both as the base for flexion actuation and as the carrier of lateral swinging components. To realize two DOFs per finger, the prototype employs two miniature motors per finger: one drives flexion–extension via the lift-ring planar linkage, and the other drives adduction–abduction via the lateral swinging mechanism. A wrist-worn module supplies power and dispatches commands to both actuators. In this study, predefined trajectory replay was used for passive/semi-passive training. A dedicated follow-up article will detail the mechatronic and control architecture.

Lightweight construction was another design focus. The transmission linkages were fabricated using 3D-printed engineering plastics, significantly reducing weight while maintaining sufficient strength and stiffness. Finite element analysis (FEA) was used to optimize link cross-sections. Results showed that under an external load of 50 N, both maximum stress and maximum deformation remained within safe limits. Compared with traditional rigid exoskeletons, the proposed structure is much lighter, making it suitable for long-term rehabilitation training.

For human–machine interfacing, a semi-open glove attachment was designed, integrating Velcro straps, magnetic locks, and finger loops for quick donning and secure fixation. This not only simplifies the wearing process but also ensures adaptability across different hand morphologies. Tests demonstrated that the fixation effectively reduced localized pressure concentrations while improving comfort.

In summary, the proposed ten-DOF exoskeleton integrates kinematic design, structural optimization, and ergonomic attachment into a unified framework. Its unique features include: achieving dual DOFs (flexion–extension and abduction–adduction) through a three-loop ten-linkage mechanism, reducing load via lightweight materials and optimized design, and aligning mechanically with human ICORs using biomimetic kinematic pairs. This device provides a lightweight, high-DOF, biomimetic solution for future rehabilitation training experiments.

## 3. Experiment and Result Analysis

### 3.1. Kinematic Experiments of the Exoskeleton

**Figure 9:**
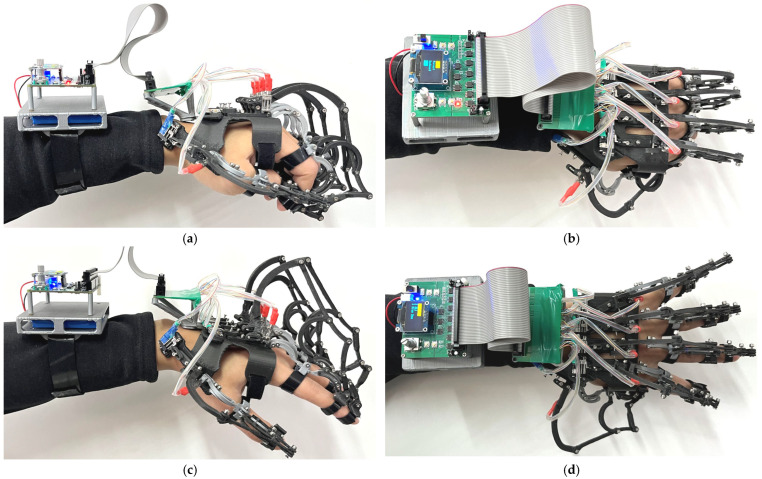
Kinematic tests of the exoskeleton (the wrist-worn module visible in the photographs is a data/power and control distribution unit). (**a**) Front view of finger flexion and adduction, (**b**) Top view of finger flexion and adduction, (**c**) Front view of finger extension and abduction, (**d**) Top view of finger extension and abduction.

To assess the kinematic consistency and reachable workspace of the proposed exoskeleton in both flexion–extension and abduction-adduction, healthy participants wore custom-fitted ten-DOF devices and performed passive, robot-driven extreme motion tests. Because the available space after donning the exoskeleton does not allow the use of the self-adaptive angle acquisition rig—and this rig is unable to measure lateral abduction/adduction—IM modules were employed for joint angle measurements, providing MCP–PIP–DIP flexion angles and inter-finger angles as primary kinematic variables. The IM modules used in this study were sensor devices manufactured by Delsys Inc. (Natick, MA, USA), providing high-accuracy joint angle measurements. The prototype employed miniature DC geared motors/short-stroke linear actuators (NIDEC, Tokyo, Japan) selected for compact size, low mass, and sufficient torque/force for the evaluated ranges of motion. A wrist-worn module integrates the power supply, motor drivers, and a microcontroller that dispatches synchronized commands to the actuators. During testing, the planar flexion linkage (lift-ring transmission) and the spatial abduction mechanism (lateral swinging ring–fixed axle) were actuated according to predefined trajectories, granting each finger a dual-DOF motion (flexion–extension plus abduction–adduction) for rehabilitation tasks, as shown in Figure 9.

For the single-finger/single-joint task, only one finger was actuated to perform either abduction–adduction or flexion–extension, and joint angles (MCP, PIP, DIP) and limits were recorded, as shown in Table 1. For the multi-finger/combined task, flexion and lateral mechanisms were co-activated to capture coordinated closing and opening. With the natural neutral posture set as 0°, and positive angles defined toward the index finger side, the measured extreme ranges in healthy fingers were approximately −20° to 25° for MCP abduction (four fingers) and ±30° for the thumb. Under exoskeleton assistance, flexion ranges reached approximately MCP 0–75°, PIP 0–103°, and DIP 0–74°. For patients with motor impairment, typical abduction ranges are about 0–45° (four fingers) and 0–60° (thumb); these limits depend on the geometry of the exoskeleton.

**Figure 10:**
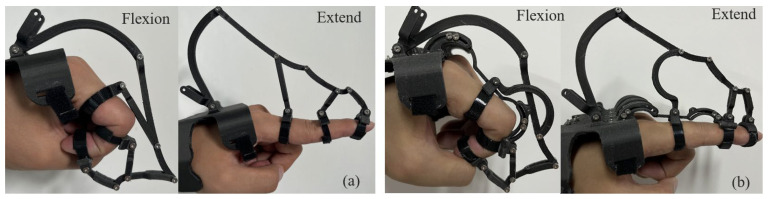
Kinematic tests of the hand exoskeleton before (**a**) and after (**b**) adding the crossing-type revolute pair.

After completing the baseline kinematic tests of the hand exoskeleton, we collected kinematic data before and after installing the crossing-type revolute pair to evaluate the effectiveness of the finger-joint kinematic model and the equivalent instantaneous center of rotation (equivalent ICOR). With the crossing-type pair installed, no interference occurred in extension; however, interference could arise during flexion with certain links. To ensure normal motion after installation, the interfering link was redesigned as a relieved/contoured (profiled) link, as shown in Figure 10. Considering the limited space and small motion range of the DIP joint, the crossing-type pair was not installed at the DIP joint in this experiment.

**Figure 11:**
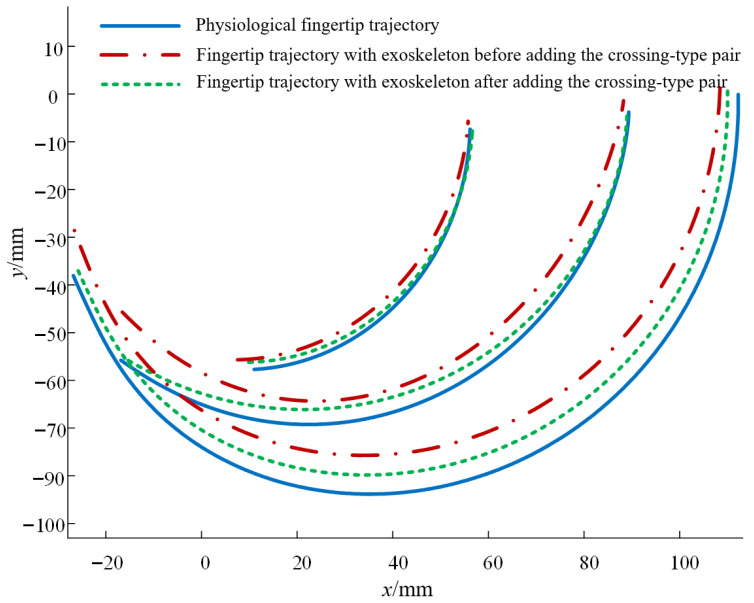
Comparison of fingertip trajectories among physiological motion, original exoskeleton, and exoskeleton with crossing-type revolute pair.

Under both conditions (with and without the crossing-type pair), 20 cycles of finger flexion–extension were performed. Joint angles were recorded and used to compute the endpoint trajectories of each joint; the average of the 20 trajectories was then used as the representative trajectory, as shown in Figure 11. The root-mean-square error (RMSE) between the joint endpoint trajectories and the physiological fingertip trajectory is summarized in Table 2.

The results indicate that after installing the crossing-type revolute pair, the joint endpoint trajectories match the physiological fingertip trajectories more closely. This demonstrates that when the exoskeleton rotates about the finger’s equivalent ICOR, its kinematic performance conforms more closely to natural finger motion, thereby validating the proposed joint kinematic model and suggesting improved human–machine compatibility of the exoskeleton designed in accordance with this model.

### 3.2. Experimental Validation of ICOR Alignment at MCP and PIP

In this study, pressure variation experiments focus on MCP and PIP, where equivalent-ICOR alignment was implemented; the DIP joint—retained as a conventional joint due to spatial constraints—is not included in the present pressure tests and is planned for future validation once a miniaturized biomimetic pair and online compensation are in place. Since the trajectory of the instantaneous center of rotation (ICOR) is short and difficult to measure directly, wearing pressure tests were conducted as an indirect method to validate the effectiveness of the biomimetic kinematic pairs in improving ICOR alignment. The underlying hypothesis is that if the exoskeleton’s rotation center is better aligned with the finger’s equivalent ICOR, relative sliding between the finger and the exoskeleton will be reduced, leading to a more uniform pressure distribution at the contact interface, characterized by lower peak and mean pressures.

The experiment was performed on the same five healthy volunteers. Each subject wore the exoskeleton without biomimetic kinematic pairs and the exoskeleton with biomimetic kinematic pairs, performing 20 cycles of finger flexion–extension in each condition. Pressure was measured using thin-film pressure sensors (Tekscan, Boston, MA, USA), which were attached to the MCP and PIP support regions in contact with the dorsal side of the hand. The sampling frequency was set at 100 Hz, and flexion angles were recorded simultaneously.

**Figure 12:**
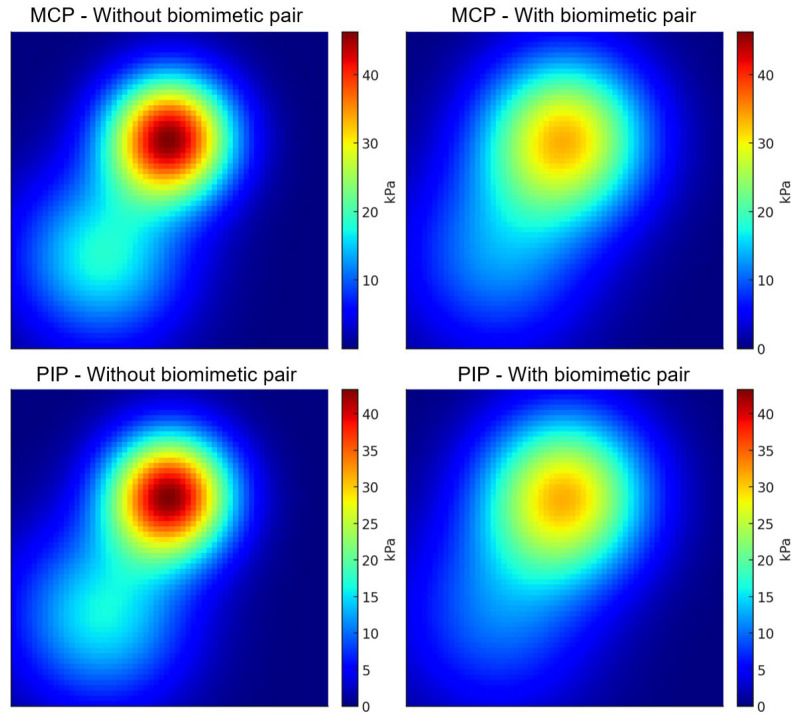
Pressure distribution heatmaps at MCP and PIP (without vs. with biomimetic kinematic pairs).

The processed data included peak pressure, mean pressure, and pressure distribution area. Results showed that with biomimetic kinematic pairs, the mean pressure at MCP and PIP decreased by approximately 15–25%, peak pressure decreased by 20–30%, and the pressure distribution became more uniform with significantly fewer local high-pressure regions. Statistical analysis (paired *t*-test, *p* < 0.05) confirmed significant differences between the two conditions, as shown in Figure 12.

These findings demonstrate that by incorporating biomimetic kinematic pairs, the exoskeleton’s rotation center was brought closer to the finger’s equivalent ICOR, significantly improving interface pressure distribution and thereby validating the effectiveness of ICOR alignment. In other words, the Instant Radius Method-based modeling and biomimetic design not only ensured kinematic consistency but also enhanced ergonomic comfort and human–machine compatibility.

## 4. Discussion

This study starts with medical imaging and proposes the Instant Radius Method (IRM) to model the continuous trajectory of the instantaneous center of rotation (ICOR) of finger joints. The modeling result was then transformed into engineering design through the framework of “equivalent ICOR—biomimetic kinematic pair—three-loop ten-linkage exoskeleton.” Around the central question—whether ICOR alignment can bring tangible benefits in human–machine matching—two independent lines of evidence were provided: kinematic consistency (joint and endpoint trajectories reconstructed by IMU [Delsys] compared with physiological trajectories), and interface pressure distribution (average and peak contact pressures at MCP and PIP decreased significantly and became more uniform). Below, we discuss the findings from four perspectives: methodological significance, structural trade-offs, errors and limitations, and future engineering/clinical implications.

### 4.1. Imaging-Driven ICOR Modeling and Engineering Significance

Conventional exoskeletons usually assume fixed rotational axes for finger joints, ignoring the rolling–sliding coupling that leads to ICOR drift. This causes persistent misalignment and shear forces during flexion–extension. The IRM proposed in this study uses CT-reconstructed articular contact curves to define the modulus and direction of the “instant radius” based on contact displacement–angle variation ratio, thus obtaining a continuous ICOR trajectory in polar coordinates. The trajectory was then condensed into an equivalent ICOR for engineering design. Unlike discrete posture-based estimation, the IRM provides continuous constraints across the entire flexion range, establishing a calculable mapping between joint geometry and linkage parameters (e.g., link lengths, arrangement angles, revolute pair positions). This mapping is valuable because it (1) enables integrated optimization of anatomical and mechanical parameters, and (2) explains and quantifies the causal chain of “misalignment → pressure concentration → discomfort,” thereby translating biomimetic design into engineering language.

The kinematic experiments confirmed that after introducing biomimetic kinematic pairs, fingertip trajectories aligned more closely with physiological ones. The pressure tests further demonstrated that geometric alignment translated into perceptible ergonomic benefits.

### 4.2. ICOR Alignment and Human–Machine Compatibility

The direct benefit of ICOR alignment is the reduction in relative sliding. When the exoskeleton’s equivalent rotation center coincides with the finger’s equivalent ICOR, tangential forces at the skin–support interface decrease, and high-pressure areas shrink. At MCP and PIP, we measured that with biomimetic kinematic pairs, mean pressure decreased by ~15–25% and peak pressure decreased by ~20–30%, while heatmaps showed dispersed pressure fields and fewer localized peaks. At the same time, IMU (Delsys)-based trajectory reconstruction showed that fingertip trajectories derived from joint angles matched physiological curves more closely. These findings jointly confirm the consistency among geometric alignment, kinematic conformity, and pressure reduction. In other words, IRM provides not only mathematical curve fitting but also empirically supported improvements in human–machine interaction.

### 4.3. Trade-Offs in Multi-DOF Linkage Exoskeleton Design

In this work, a lateral swinging mechanism was added on top of the planar flexion structure, enabling each finger to achieve dual DOFs (flexion–extension + abduction–adduction), resulting in ten DOFs across five fingers. Biomimetic kinematic pairs were added to MCP and PIP to follow equivalent ICORs. The three-loop ten-linkage architecture balances reachability, stiffness, and weight: the first loop provides primary actuation, the second loop corrects trajectories for alignment, and the third loop provides lateral DOFs.

Analysis in Lagrangian coordinates showed only nine independent coordinates out of eleven, indicating an underactuated system. Underactuation helps reduce actuator size and overall weight but also increases coupling and tuning complexity. To avoid mutual interference between flexion and lateral DOFs, the flexion platform and lateral fixed shaft were designed with structural decoupling and travel limits. Links prone to interference were reshaped into profiled links to ensure continuous and reliable motion even at extreme postures. At the DIP joint, due to spatial limitations and small motion amplitude, conventional revolute pairs were retained. This reflects a trade-off between alignment benefits and structural complexity.

In this work, biomimetic kinematic pairs were implemented at MCP and PIP to follow equivalent ICORs, while the DIP retained a conventional revolute pair due to spatial constraints and small motion amplitude. This choice preserves compactness and robustness but introduces a trade-off in ‘full-finger alignment’: improvements in fingertip trajectory and interface pressure are concentrated at MCP/PIP, with a small residual at the distal end. In addition to reshaping interference-prone links into profiled links to ensure reliable motion at extreme postures, future efforts will focus on miniaturizing a DIP biomimetic pair and enabling online alignment compensation to further enhance distal consistency.

### 4.4. Sources of Error and Methodological Limitations

Several error sources and limitations should be noted: (1) Imaging and modeling error: CT scans were acquired at quasi-static key postures. Although fiberglass splints were used for rapid fixation, discrepancies may exist compared with dynamic natural motion. Thresholding/region-growing segmentation and curve fitting may introduce local errors, especially on the phalangeal base side, where scattered points required piecewise fitting. (2) Mechanical approximation error: The equivalent ICOR approximates a continuous trajectory by a single center, which is a “global optimal—local compromise.” Manufacturing tolerances, backlash in lead screw drives, and bearing friction may reduce alignment accuracy. (3) Measurement error: IMUs are subject to drift from gyroscope integration and magnetic disturbances; thin-film pressure sensors are sensitive to surface curvature and shear, making inter-subject comparisons less robust. (4) Sample and task limitations: Modeling was based on healthy volunteers; validation was performed under passive mode and typical rehabilitation tasks. More complex conditions—pathological variability, muscle tone changes, active–passive coordination—were not addressed. (5) Structural limitations: The DIP joint did not receive a biomimetic kinematic pair, limiting complete alignment. The thumb’s coupled motions were also not fully covered by the current architecture.

### 4.5. Engineering and Clinical Implications

Despite these limitations, this study presents a closed-loop methodology from imaging → modeling → mechanism design → experimental validation. Future work may proceed along three directions: (1) Personalized parameter identification: Employ CT/MRI, low-dose CBCT, or ultrasound combined with IRM-based fitting to derive patient-specific equivalent ICORs and linkage calibration. (2) Full-finger alignment: Extend biomimetic pairs to the DIP and thumb CMC/MCP joints with miniaturized designs and relieved/adaptable modules to balance compactness with accuracy. (3) Multi-sensor fusion and closed-loop control: Integrate IMU, force/torque, and surface EMG signals to enable force–position hybrid control and adaptive alignment compensation, adjusting misalignment online during rehabilitation training. To achieve full-finger alignment, we plan to extend biomimetic pairs to the DIP joint with miniaturized and relieved/compliant modules to balance compactness and accuracy. In parallel, by fusing IMU, force/torque, and sEMG signals, we will develop force–position hybrid closed-loop alignment compensation to reduce residual misalignment online during rehabilitation training. We initialize using population-prior/contralateral ICOR templates with a brief ROM calibration (optionally low-dose extremity imaging), use early bedside assessments to forecast near-term function and stratify the initial prescription, and perform weekly micro-adjustments guided by wearable IMU/pressure/EMG monitoring under conservative force–position limits to progressively increase intensity and task complexity.

Clinically, the next step is to conduct multi-center randomized controlled trials comparing “aligned vs. non-aligned” exoskeletons in terms of pain, comfort, range of motion, FMA-UE, and ARAT scores, as well as long-term adherence and usability in home settings. From an industrial perspective, the combination of lightweight 3D-printed engineering plastics and limited aluminum alloy parts, modular actuation, and quick-donning human–machine interfaces (flexion platform, Velcro/magnetic locks/finger loops) provides realistic feasibility for cost control and scalability.

In summary, the IRM offers a continuous and engineerable description of finger joint kinematics; the “equivalent ICOR—biomimetic kinematic pair—three-loop ten-linkage” framework translates this description into mechanism design. Both kinematic and pressure-based evidence demonstrate that ICOR alignment significantly improves human–machine compatibility. With further advances in personalization, full-finger alignment, and closed-loop control, this approach holds strong potential for enhancing rehabilitation training and functional assistance.

## 5. Conclusions

This study proposed the Instant Radius Method (IRM) to continuously model the kinematics of finger joints based on medical imaging and subsequently designed a lightweight ten-DOF exoskeleton equipped with biomimetic kinematic pairs. A closed-loop workflow was established, covering modeling, mechanism design, and experimental validation. The main conclusions are as follows: (1) Finger joint kinematic modeling: The IRM was introduced to extract articular contact points from CT images and transform them into polar coordinate-based instant radius functions, thereby obtaining continuous ICOR trajectories for the MCP, PIP, and DIP joints. An equivalent ICOR model was constructed for engineering application, providing a more physiologically consistent description of joint motion. (2) Biomimetic kinematic pairs and exoskeleton design: Based on the equivalent ICOR, crossing-type biomimetic kinematic pairs were developed and integrated with a three-loop ten-linkage structure, resulting in a lightweight exoskeleton with dual DOFs per finger (flexion–extension and abduction–adduction, ten DOFs in total). The design ensured compactness and light weight while aligning the exoskeleton’s rotation centers with human joints. (3) Kinematic validation: Experiments using IMU sensors (Delsys) demonstrated that after adding the biomimetic kinematic pairs, joint angles and fingertip trajectories generated by the exoskeleton matched physiological trajectories more closely, with significantly reduced RMSE values. (4) Pressure distribution validation: Thin-film pressure sensor tests confirmed that biomimetic kinematic pairs significantly improved interface pressure distribution. At the MCP and PIP joints, mean pressure decreased by approximately 15–25% and peak pressure decreased by 20–30%, with fewer localized high-pressure regions, thereby demonstrating the effectiveness of ICOR alignment in enhancing comfort and human–machine compatibility.

In summary, through the integrated pathway of IRM-based modeling → biomimetic kinematic pair → multi-DOF exoskeleton design → experimental validation, this work demonstrated the importance and effectiveness of ICOR alignment in hand rehabilitation robotics. Future research will extend toward personalized customization, full-finger alignment, and closed-loop control, paving the way for clinical application and functional assistance.

## Figures and Tables

**Table 1:** Finger joint angle range with hand rehabilitation robot assistance.

Testing Method	Finger	MCP Joint Abduction/Adduction (°)	MCP Joint Flexion/Extension (°)	PIP Joint Flexion/Extension (°)	DIP Joint Flexion/Extension (°)
Single motion	Four fingers	−20~25	0~75	0~103	0~74
	Thumb	±30	0~75	-	0~78
Combined motion	Four fingers	−14.2~19.7	1.3~74.2	2.7~97.5	0.8~63.2
	Thumb	−20.5~21.8	0~74.5	-	0~78.6

**Table 2:** RMSE (mm) of exoskeleton trajectories compared with physiological fingertip trajectories.

Mechanism Type	MCP Joint Trajectory	PIP Joint Trajectory	DIP Joint Trajectory
Original exoskeleton mechanism	1.532	5.764	7.259
Exoskeleton with crossing-type revolute pairs	0.725	1.271	2.364

## Data Availability

Details of data availability are available from the first author on request.
